# Association between Neutrophil-to-Lymphocyte Ratio with Abdominal Obesity and Healthy Eating Index in a Representative Older Spanish Population

**DOI:** 10.3390/nu12030855

**Published:** 2020-03-23

**Authors:** Elena Rodríguez-Rodríguez, Ana M. López-Sobaler, Rosa M. Ortega, M. Luisa Delgado-Losada, Ana M. López-Parra, Aránzazu Aparicio

**Affiliations:** 1 Department of Chemistry in Pharmaceutical Sciences, Analytical Chemistry, Faculty of Pharmacy, Complutense University of Madrid, 28040 Madrid, Spain; elerodri@ucm.es; 2 UCM Research Group: VALORNUT-920030, Department of Nutrition and Food Science, Faculty of Pharmacy, Complutense University of Madrid, Plaza Ramón y Cajal S/N, 28040 Madrid, Spain; asobaler@ucm.es (A.M.L.-S.); rortega@ucm.es (R.M.O.); mldelgad@ucm.es (M.L.D.-L.); 3 Department of Nutrition and Food Science, Faculty of Pharmacy, Complutense University of Madrid, Plaza Ramón y Cajal S/N, 28040 Madrid, Spain; 4 Department of Experimental Psychology, Psychological Processes and Speech Therapy, Faculty of Medicine, Complutense University of Madrid, Plaza Ramón y Cajal S/N, 28040 Madrid, Spain; 5 Department of Legal Medicine, Psychiatry and Pathology, Faculty of Medicine, Complutense University of Madrid, Plaza Ramón y Cajal S/N, 28040 Madrid, Spain; amlopezparra@med.ucm.es

**Keywords:** abdominal obesity, inflammation, neutrophil-to-lymphocyte ratio, older adults, healthy-eating index

## Abstract

Poor diet quality and obesity, especially abdominal obesity, have been associated with systemic inflammation. The neutrophil-to-lymphocyte Ratio (NLR) is an available and inexpensive inflammation biomarker. The aim of the present study was to determine the association of dietary patterns and obesity with an inflammatory state. A group of 1747 Spanish noninstitutionalized older adults individuals were included, and a food-frequency questionnaire was applied. The Global Food Score (GFS) and Healthy Eating Index for Spanish population (SHEI) were calculated. Weight, height and waist (WC) and hip circumferences were measured, and BMI, waist-to-height ratio (WHtR), and waist-to-hip ratio (WHR) determined. In addition, body-fat percentage was measured by bioimpedance. NLR was calculated (NLR ≥ p80: 2.6; 2.8 and 2.4 as inflammatory status in the entire population, men and women, respectively). The men with inflammatory status presented significative higher values of WC, WHtR, WHR, and body-fat percentage (101.82 ± 10.34 cm, 0.61 ± 0.06, 0.98 ± 0.06, and 31.68 ± 5.94%, respectively) than those with better inflammatory status (100.18 ± 10.22 cm, 0.59 ± 0.06, 0.97 ± 0.07, and 30.31 ± 6.16%, respectively). Those males with worse inflammatory state had lower scores for protein foods (OR = 0.898 (0.812–0.993); *p* = 0.037). The women with NLR ≥ 2.4 had higher WHtR and WHR (0.62 ± 0.09 and 0.91 ± 0.09) than those with NLR < 2.4 (0.60 ± 0.08 and 0.90 ± 0.08). In multiple linear regression analysis, NLR was positively related with WHtR and negatively related with SHEI score (β = 0.224 ± 0.094; *R*^2^ = 0.060; *p* < 0.05 and β = −0.218 ± 0.101; *R*^2^ = 0.061; *p* < 0.05), adjusting by sex, age, marital status, education level, smoking, hours of sleeping and inflammatory diseases. In women, the higher the SHEI and GFS scores were and the better meeting the aims of cereal and vegetable servings, the less the odds of inflammatory status (OR = 0.970 (0.948–0.992); *p* = 0.008; OR = 0.963 (0.932–0.995); *p* = 0.024; OR = 0.818 (0.688–0.974); *p* = 0.024 and OR = 0.829 (0.730–0.942); *p* = 0.004, respectively). WHtR and quality of diet is related to the inflammation status in older adults regardless to the sex.

## 1. Introduction

Healthy dietary patterns, due to their anti-inflammatory effects, have been linked to their protective effect on chronic diseases. For example, a high adherence to a Mediterranean dietary pattern (MDP), which is considered healthy and prudent, has been associated with a better health status due to the protective effect that this pattern shows against various chronic diseases [1,2,3], including a favorable effect on total mortality, cardiovascular disease, and several cancers [3,4,5]. The MDP has also been proposed as a determinant of the longevity of these populations [6]. All these beneficial effects could be due to its anti-inflammatory effect [7], related to its high content of vegetables, legumes, fruits and nuts, cereals, fish, and olive oil, and low content of saturated lipids, meat, and poultry [8,9]. Similarly, other studies observed that a high intake of trans fatty acids has a positive correlation with inflammation [10], a diet high in meat and processed food is positively correlated with inflammation [11,12], and, in contrast, a high intake of vegetables is inversely associated with C-reactive protein (CRP) concentrations [13,14].

Obesity is also associated with inflammation. A previous study revealed that obesity defined by body-mass index (BMI) and waist circumference (WC) was associated with inflammation [15]. Body fat, skinfold thickness, and other measures of abdominal adiposity were also positively correlated with inflammation [16,17]. Although systemic inflammation is produced in obesity as a consequence of excessive adipose-tissue accumulation, it is generally related to deleterious health outcomes [15,18,19,20]. Obesity-related adverse health consequences occur predominately in individuals with upper body fat distribution that is commonly associated with increased central adiposity [21,22,23].

Neutrophils and lymphocytes constitute the first line of defense against infection and play a major role in inflammation. The neutrophil-to-lymphocyte ratio (NLR), determined as the ratio of absolute neutrophil count to lymphocyte count, is a novel inflammatory biomarker used as a prognostic factor in various diseases [24]. It has been previously studied [25,26,27,28], but in light of the racial and ethnic differences found in NLR [29], more studies examining these relationships in different populations should be conducted. Although several studies have investigated the effect of dietary patterns, anthropometric measurements, or metabolic parameters on inflammatory markers, studies using the NLR as an inflammation indicator are not common.

Thus, the objective of this study was to determine the association of dietary patterns and abdominal obesity with inflammatory markers in a noninstitutionalized Spanish older adult population.

## 2. Materials and Methods

### 2.1. Study Design

The pilot survey of the Aging in Spain Longitudinal Study (ELES Study) is a cross-sectional study conducted in Spain, designed to collect health variables, food habits, socioeconomic data, and cognitive and functional capacities to analyze the aging process. The ELES study was carried out on a Spanish representative sample of noninstitutionalized persons of 50 years or older. Details of the study were published elsewhere [30,31].

All participants gave their informed consent for inclusion before they participated in the study. The study was conducted in accordance with the Declaration of Helsinki, and the protocol was approved by the Ethics Committee of Hospital Clínico San Carlos (ref. 17/125-E).

### 2.2. Subjects

This observational study was conducted in 2011. Sampling was representative of noninstitutionalized people over 50 years (*n* = 1747; 771 men and 976 women).

Population censuses from January 2009 collected by the National Statistics Institute of Spain were the source of information for the census sections. From 15.4 million people, approximate three-stage cluster sampling was carried out (municipality census section; household; household member), with stratification of the first-stage units depending on the habitat size of the municipality to which they belonged (<10,001 inhabitants; between 10,001 and 100,000; between 100,001 and 500,000; >500,001). Other stratification variables were sex (male, female) and age (50–59; 60–69; 70–79; and 80 years and older). Data collection was performed in two parts: The first part consisted of a computer-assisted telephone interview (CATI) in which personal data (sex and age) were collected. The second part of the study consisted of a nurse visit who took the blood samples and anthropometric measurements and performed the Mini Mental-State Examination [32] and a computer assisted personal interview (CAPI). If the scores of a specific part of the Mini-Mental reached pre-established thresholds, there was a self-administered questionnaire asking for marital status, educational level, and smoking habits, among other variables, in addition to a food-frequency questionnaire. This visit was followed by another to collect the self-administered questionnaires.

### 2.3. Food Consumption

Dietary assessment was based on a quantitative food-frequency questionnaire (FFQ). It included 40 questions about food groups and selected food items (e.g., skimmed milk, chocolate, and processed food) [33].

The consumption adequacy of the 5 food groups (Global Food Score, GFS) was determined following the same methodology followed by Kennedy et al. [34] for the food groups items from the original Healthy Eating Index (HEI): Cereals, fruits, vegetables, dairy, and protein foods. Each item scored from 0 to 10 points, where 10 meant that the recommendation of the different food groups was correctly met, and 0 that the recommendation was not met at all. The sum of the scores for each variable resulted in this GFS, with a maximum of 50 points being obtainable.

Having in mind that the complete HEI could not be determined from the FFQ in this study, a Healthy Eating Index adapted for Spanish population (SHEI) was determined [35] using the methodology proposed by Norte and Ortiz [36], and Hernández and Goñi [37] for Spanish people. All food variables were classified into 10 groups: Cereals, vegetables, fruits, dairy products, fats and oils, meats, legumes, processed meats, sweets, and sauces and processed food (Table 1). These variables were divided into 5 categories that refer to the frequency of consumption: 1, daily; 2, three or more times a week, but less than daily; 3, once or twice a week; 4, less than once a week; and 5, never or almost never. The new variables were categorized as follows: the first 5 represent daily-consumption food groups, 6 and 7 correspond to the weekly-consumption food groups, and the rest represent the occasional-consumption food groups.

The SHEI score was determined by adding the individual scores obtained in each food group, with a theoretical maximal score of 100 points. SHEI score was classified into three categories: Healthy (>80 points), need changes (>50 and ≤ 80 points), and inadequate diet quality (<50 points). To set the cut-off points of the new index, and to interpret and operationally define the concepts of “healthy”, “need changes”, and “unhealthy”, various empirical studies were reviewed [34,36,37,38,39]; helping the research team to reach a consensus was one of the compelling reasons to maintain the original cut-off points [36,37,38,39], as well as to compare the obtained results in this work with other studies at the international level.

### 2.4. Body Measurements

Anthropometric measurements (weight, height, and WC and hip circumferences) were individually obtained by trained interviewers following standardized procedures [40].

Weight was measured in participants wearing light clothing or underwear using a Tefal Sense PP 1027 portable weighing scale (range, 0.1–160 kg; precision, 100 g). Height was assessed in triplicate using a PSYMTEC mod. 601 anthropometer (range, 0–200 cm; precision, 1 mm). WC and hip measurements were determined using Holtain flexible metallic tape (range, 0–150 cm; precision, 1 mm). For these measurements, participants were barefoot and wore only underwear.

General obesity was assessed using body-mass index (BMI), and abdominal obesity was assessed using WC, waist-to-height ratio (WHtR), and waist-to-hip ratio (WHR).

BMI was determined as weight (kg)/height^2^ (m^2^). According to the BMI, participants were grouped as individuals with underweight: BMI < 22 kg/m^2^; normal weight: BMI: 22–27 kg/m^2^; obesity: BMI > 27 kg/m^2^ [41].

High WC (abdominal obesity) was defined as ≥80 cm for women and ≥94 for men [42]. WHtR was determined as WC (cm)/height (cm). Haung et al. [43] proposed that those female participants with WHtR ≥ 0.53 and those men with WHtR ≥ 0.52 had abdominal obesity. On the other hand, WHR was also determined as: WC (cm)/hip circumference (cm). Participants were classified into two categories: those with abdominal obesity (WHR > 0.85 for women and >1 for men), and those without abdominal obesity (WHR ≤ 0.85 for women and ≤ 1 for men) [44].

Body-fat percentage was determined through noninvasive bioimpedance analysis with an Omron BF306 body-composition monitor. According to Bray and Popkin criteria [45], participants were classified into three categories: normal weight (body-fat percentage: 20–30% for women and 12–20% for men), overweight (body-fat percentage: 31–33% for women and 21–25% for men), and obesity (body-fat percentage: >33% for women and > 25% for men).

### 2.5. Biochemical Variables

Blood samples were collected after overnight fasting for 12 h. A Model S Coulter Counter (Coulter Electronic Limited, Luton, UK) was used to determine the complete blood cell counts and differential counts of white blood cells in anticoagulated whole blood [46]. The neutrophil-to-lymphocyte ratio (NLR) was determined using absolute neutrophil count divided by absolute lymphocyte count. The definition of inflammation in this study was established according to the 80 percentile (p80): NLR ≥ 2.6 for the entire population, NLR ≥ 2.8 for men, and NLR ≥ 2.4 for women.

### 2.6. Other Variables

Potential covariates for adjustment included demographic and health information (e.g., age, education level, marital status, smoking status, sleeping hours, and inflammatory diseases). Marital status was categorized as single, married, in a couple, widow or widower, and divorced. Education level was categorized as very low (do not know to read or write), low (high school and below), and high (above high school). Smoking status was categorized as nonsmoker, ex-smoker, daily smoker, and not daily smoker. Inflammatory disease was categorized as yes or not considering if the participants had: Arthrosis, asthma, chronic obstructive pulmonary disease, and diabetes or not.

### 2.7. Statistical Analysis

The results for all variables are expressed as mean ± standard deviation (SD) or percentages, where appropriate. For non-normal variables, the median and the interquartile range are also presented. The Kolmogorov–Smirnoff test was used to test whether the variables followed normal distribution to decide between parametric or nonparametric analysis. Student’s *t* test or the Mann–Whitney U test, depending on whether or not data were normally distributed, was used to examine differences between NLR and sex groups. When comparing the proportions, the *z*-test was used.

Relationships between NLR and dietetic and anthropometric data were examined by Pearson’s correlation. Relationships between NLR and dietetic and anthropometric data were examined by linear and multiple regression analysis, including covariates. Not normal distribution variables were log transformed. The odds ratios (OR) with 95% confidence interval was derived using multivariate logistic regression analysis to compare the association of dietary patterns, anthropometric status, and metabolic parameters with NLR levels in men and women. We have multiplying by 10 variables with decimal fractions (WHR, WHtR) and the chance of inflammation occurring by a change of 0.1 is obtained in the odds ratio.

Two models were performed in linear and multiple regression analysis. Model 1 was unadjusted, Model 2 was adjusted for sex (only in linear analysis), age, marital status, education level, smoking, hours of sleeping and inflammatory diseases (arthrosis, asthma, chronic obstructive pulmonary disease, and diabetes). A significance level of *p* ≤ 0.05 was used for all analyses. Statistical analyses were performed using statistical software package SPSS, version 25.0, for Windows.

## 3. Results

The mean age of the sample was 66.2 ± 10.64 years, and a sex-related difference was seen at the age of the study participants. Mean SHEI was 76.52 ± 8.38, and mean NLR was 2.03 ± 0.89, with differences between men and women (75.08 ± 8.54 vs. 77.67 ± 8.08 points, *p* < 0.001; and 2.15 ± 0.96 vs. 1.93 ± 0.82, *p* < 0.001, respectively) (Table 2). Mean WC was 97.41 ± 12.12 cm, and mean WHtR was 0.60 ± 0.08, and also there were differences between men and women (100.67 ± 10.42 vs. 94.79 ± 12.75 cm; *p* < 0.001, and 0.60 ± 0.06 vs. 0.61 ± 0.09; *p* < 0.01, respectively) (Table 3).

Males with a worse inflammatory state (NRL ≥ 2.8) had higher values of anthropometric parameters related to the presence of central obesity (WC, WHtR, and WHR) than those with a lower inflammatory state. In addition, those males with inflammatory status had lower punctuations for protein foods (5.60 ± 2.22) than those with worse inflammatory status (6.10 ± 2.29; *p* < 0.05). Women with the worst inflammatory state (NRL ≥ 2.4) also had higher WHtR and WHR values than those with a lower inflammatory state, and also had the former, a lower GFS and SHEI score value than the latter (Table 4 and Table 5). Furthermore, those women with worse inflammatory status had lower scores for vegetables (2.62 ± 1.44) than those with better inflammatory status (3.02 ± 1.60; *p* < 0.05).

Inverse correlation was found between NLR and SHEI (β = –0.007 ± 0.003; *r* = 0.060; *p* < 0.05), and positive correlation between NLR and age (β = 0.016 ± 0.002; *r* = 0.192; *p* < 0.001), BMI (β = 0.011 ± 0.005; *r* = 0.057; *p* < 0.05), WC (β = 0.007 ± 0.002; *r* = 0.100; *p* < 0.001), and WHtR (β = 1.119 ± 0.323; *r* = 0.096; *p* < 0.001).

In multiple linear regression analysis, NLR was positively related with WHtR and negatively related with SHEI score (unadjusted model) (β = 0.338±0.087; *R*^2^=0.011; *p* < 0.001 and β = −0.199 ± 0.100; *R*^2^ = 0.003; *p* < 0.05) and (β = 0.224 ± 0.094; *R*^2^ = 0.060; *p* < 0.05 and β= −0.218 ± 0.101; *R*^2^ = 0.061; *p* < 0.05) (adjusted model by covariates such as age, marital status, educational level, smoking, sleep duration, and inflammatory diseases).

**Figure 1:**
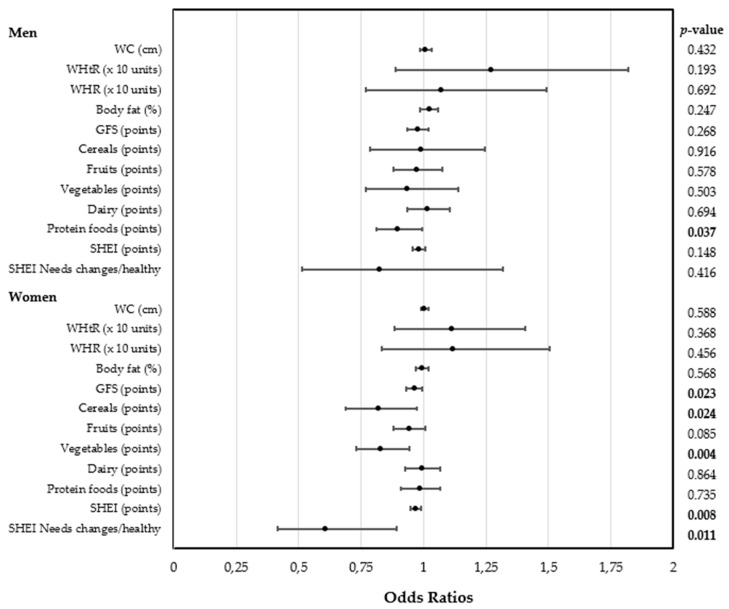
Odds ratio for high neutrophil-to-lymphocyte ratio by different anthropometric and dietetic data. WC, waist circumference; WHtR, waist-to-height ratio; WHR, waist-to-hip ratio; GFS, Global Food Score; SHEI, Spanish Healthy Eating Index.

Setting NLR ≥ 2.8 (p80) as the cut-off point to define inflammation in men, it was observed that those men with worse inflammatory status had higher body-fat percentage and WHtR, indicative of abdominal obesity, than those with better inflammatory status (Table 4). In particular, the higher the values of body-fat percentage and WHtR were, the greater the odds of developing a high inflammatory state was (unadjusted model) (OR 1.037 (1.001–1.074), *p* = 0.042; and OR 1.610 (1.151–2.251), *p* = 0.005, respectively). Signification was lost when adjusted by covariates (Figure 1). However, it was found that those males with worse inflammatory state had lower scores for protein foods (unadjusted model: 0.905 (0.823–0.997); *p* = 0.043, adjusted model by covariates: 0.898 (0.812–0.993); *p* = 0.037). In addition, in women, the higher SHEI and GFS scores were, the less the odds to present a high inflammatory state was in unadjusted model (OR 0.971 (0.950–0.993), *p* = 0.010; and OR 0.968 (0.937–0.999), *p* = 0.010, respectively) and adjusted model by covariates (OR 0.970 (0.948–0.992), *p* = 0.008; and OR 0.963 (0.932–0.995), *p* = 0.023, respectively) (Figure 1). Furthermore, following more appropriate diets (with a SHEI score > 80 points) was a protective factor against a high inflammatory state (NLR ≥ 2.4) in unadjusted model and adjusted model by covariates (OR 0.636 (0.438–0.923); *p* = 0.017 and OR 0.609 (0.416–0.891); *p* = 0.011, respectively). Similarly, achieving the objectives of cereal and vegetable servings was also a protective factor for a high inflammatory state in unadjusted model (cereals. OR 0.846 (0.714–1.003); *p* = 0.054; vegetables: OR 0.837 (0.737–0.950); *p* = 0.006) and adjusted model by covariates (cereals: OR 0.818 (0.688–0.974); *p* = 0.024; vegetables: OR 0.829 (0.730–0.942), *p* = 0.004).

## 4. Discussion

According to the obtained results, abdominal obesity is associated with a higher inflammatory state, whereas diet quality, measured by SHEI score, is associated with a lower inflammatory state.

Systemic inflammation can be measured by using a variety of biochemical and hematological markers. The local inflammatory milieu of visceral adipose tissue is characterized by monocyte/macrophage infiltration and a diversity of lymphocyte subtypes [47,48]. Thus, the total white-blood-cell count (WBC) and its subtypes could reflect an inflammatory status in the absence of infection [49]. Although novel disease-specific biomarkers have been identified, most of them are time-consuming and expensive to evaluate [50]. Observational studies have thoroughly investigated the role of CRP and total leukocyte count in different chronic conditions [50]; recently, the NLR has been incorporated as a marker of inflammation, believed to reflect the balance between innate (neutrophils) and adaptive (lymphocytes) immune responses. Previous research showed that an elevated NLR is associated with the increased concentration of various proinflammatory cytokines [51,52] that may cause cellular DNA damage. However, although research studied the relationship between this parameter and various chronic diseases with inflammation [53,54,55,56,57], there are few papers on its relationship with obesity and the quality of the diet.

In this study, we defined an inflammatory state according to an NLR value of 2.4 for women and 2.8 for men, corresponding to the p80, with an inflammatory state defined when values greater than that cut-off point were obtained. Different criteria were used in the literature to establish such a cutting point. While some studies categorized their patients according to NLR intervals (e.g., tertiles, quartiles, quintiles) [58,59,60], other studies used definite NLR cutoff points (e.g., NLR ≥ 2.5 [61], NLR ≥ 2.7 [62], NLR ≥ 3 [12,63], NLR ≥ 4 [64], and NLR ≥ 5 [65,66,67]). In the present paper, the average value of the obtained NLR from the participants was 2.03 ± 0.89, this value being higher in males than in females, which coincided with other studies where participants with high levels of inflammatory markers were more frequently men than women [12,68]. In addition, this parameter was positively related to age, which was also observed in other studies in which both NLR and various proinflammatory adipokines were shown to increase with age [69,70].

When studying the relationship of NLR to diet quality, we found that, in the general population regardless of sex, the follow-up of lower-quality diets was associated with higher inflammatory states, determined by the NLR. In this way, several studies showed that a high-quality dietary intake, characterized using the Dietary Approaches to Stop Hypertension (DASH) index, the Healthy Eating Index (HEI), and the Mediterranean Diet Index, has high anti-inflammatory potential [9,71]. In particular, our results are in line with those found by Sudera et al. [72] who, after studying 73 males and 161 females between 18 and 65 years old living on the Balearic Islands, found that low adherence to the MDP was directly associated with a worse profile of plasmatic inflammation markers. Specifically, higher adherence to the MPD was associated with higher levels of adiponectin, and lower levels of leptin, TNF-α, PAI-1, and hs-CRP in the mentioned study. Although some studies did not find differences between men and women in the effects of a prudent diet or MDP on systemic inflammation [12,73], in our study, women who followed more suitable diets (with a score higher than 80 points) were protected against a high inflammatory state (NLR ≥ 2.4), a result that was not observed in males. This could be explained by the fact that only women who had an adequate consumption of vegetables and cereals had a more adequate inflammatory state. In fact, vegetables and whole grains are foods rich in fiber and flavonoids, among other nutrients, and a number of dietary-intervention studies provided evidence that dietary fiber and flavonoids are capable of modulating inflammatory cytokines (e.g., TNF-α) and CRP production [74,75,76,77,78,79,80]. This result is highlighted because, in general, the consumption of vegetables and cereals is usually below than the recommended in the older adults Spanish population [81,82], and to enhance its consumption would improve the inflammatory state of an organism that has deteriorated with age. In men, although not effect was seen between following more suitable diets and the inflammatory state, those men with an adequate consumption of protein foods had a more adequate inflammatory state. Instances of higher overall inflammatory status, such as those of older individuals, may lead to an increased need for substrates (i.e., protein) to support anti-inflammatory processes and an adequate protein intake could be beneficial to improve this situation. In the Framingham Heart Study Offspring cohort [83], overall inflammation increased less in those with the highest quartile of protein intake (Q4: 95.9 g/day) than in those with the lowest quartile (Q1: 67.4 g/day). Nevertheless, in the mentioned study, when protein was considered by its dietary source, plant protein, but not animal protein, showed favorable associations with changes in both overall inflammation [83]. However, apart from the nature of the protein, benefits of protein in aging may depend on underlying inflammation status. In this way, protein intake was associated with decline in muscle strength in persons with high levels of inflammatory markers in a cohort of 598 older adults [84], so enhance the consumption of protein foods, would improve the inflammatory state in aging.

By analyzing the relationship between anthropometric parameters and the inflammatory state, a positive and significant correlation was found between NPL and WHtR. In addition, both males and women with worse inflammatory status had higher WHtR and WHR values (Table 5). However, in males a higher percentage of body fat and abdominal obesity was observed in males with a worse inflammatory state, although this significance was only observed in the unadjusted model. This result was not found in women, which coincided with existing data that indicate sex-specific differences existing in obesity-induced inflammation [85]. With respect to inflammation and obesity, 70–80% of obese individuals present a structural and functional reshaping of adipose tissue that causes an inflammatory reaction. When the resolution of acute inflammation is not correctly resolved, a chronic low-grade inflammatory state at the local level is triggered, and lipoinflammation occurs [86].

In this way, chronic inflammation has emerged as one of the key physiological mechanisms that links obesity with different associated pathologies, such as diabetes, cardiovascular disease, cancer, and asthma; thus, it is important to determine the presence of inflammation in a population. In particular, obesity has been linked to high CRP levels [87,88,89] and changes in the leukocyte profile [90,91]. In addition, according to our results, in a study carried out in 26,016 middle-aged and older adults (>35 years old) with metabolic syndrome in Taiwan, obesity, high body fat, high WC or hip circumference, and high WHtR were significantly associated with increased odds ratios of high CRP and NLR [12]. Furthermore, some studies in obese adults showed that weight loss improved inflammation parameters [91,92]. Nevertheless, in some previous studies, increased BMI was associated with total WBC and individual subtypes, but not with NLR [92,93,94]. More research is needed to clarify this relationship.

This study has some limitations. Because it is a cross-sectional study, causal inferences cannot be made, and the possibility of residual or unknown confounding cannot be excluded. Additionally, our study only represented a single ethnicity, and future studies should examine these relationships in other populations. Another limitation of the study is that the FFQ relies on the participant’s ability to recall and report dietary intake. In addition, the FFQ used in the ELES’s study has not been validated for Spanish people. Nevertheless, Affret et al. [95] concluded that to use a 44 items FFQ was sufficient to assess the overall diet and to describe major food and nutrient intakes. In contrast, one of the major strengths of this study is its representativeness of noninstitutionalized Spanish persons of 50 years or more, and its large subject-sample size. Moreover, anthropometric data were collected by trained staff, and they were not self-reported by the participants, which improved the validity of the study.

## 5. Conclusions

Inflammatory state, measured by the NLR, is positively associated with the presence of abdominal obesity, measured by the WHtR, and negatively associated with diet quality, measured by the SHEI score, regardless to the sex. Nevertheless, in a more detailed logistic regression analysis differentiating by sexes, following more suitable diets (with a score higher than 80 points) and to have an adequate consumption of vegetables and cereals in women and of protein foods in men, were protected against a high inflammatory state. Because an inflammatory status has been linked to different chronic diseases, it is necessary to take appropriate measures to decrease abdominal obesity and improve the diet quality.

## Figures and Tables

**Table 1:** Criteria for defining score of each food group for Spanish Healthy Eating Index (SHEI) based on methodology proposed by Norte and Ortiz (2011), and Hernández and Goñi (2015).

Variable	Criteria for 10 (Perfect) Score	Criteria for 7.5 Score	Criteria for 5 Score	Criteria for 2.5 Score	Criteria for 0 (Minimal) Score
**Daily consumption**					
Cereals	Daily consumption	3 or more servings a week, but less than daily	1 or 2 servings a week	Less than one serving a week	Never or almost never
Vegetables	Daily consumption	3 or more servings a week, but less than daily	1 or 2 servings a week	Less than one serving a week	Never or almost never
Fruits	Daily consumption	3 or more servings a week, but less than daily	1 or 2 servings a week	Less than one serving a week	Never or almost never
Dairy	Daily consumption	3 or more servings a week, but less than daily	1 or 2 servings a week	Less than one serving a week	Never or almost never
Fats and oils	Daily consumption	3 or more servings a week, but less than daily	1 or 2 servings a week	Less than one serving a week	Never or almost never
**Weekly consumption**					
Meat	1 or 2 servings a week	3 or more servings a week, but less than daily	Less than one serving a week	Daily consumption	Never or almost never
Legumes	1 or 2 servings a week	3 or more servings a week, but less than daily	Less than one serving a week	Daily consumption	Never or almost never
**Occasional consumption**					
Processed meat	Never or almost never	Less than one serving a week	1 or 2 servings a week	3 or more servings a week but not daily	Daily consumption
Sweets (desserts, honey, cocoa, etc.)	Never or almost never	Less than one serving a week	1 or 2 servings a week	3 or more servings a week but not daily	Daily consumption
Other: sauces, snacks, and prepared dishes	Never or almost never	Less than one serving a week	1 or 2 servings a week	3 or more servings a week but not daily	Daily consumption

In SHEI calculation, each variable scored from 0 to 10 points according to the criteria set out in Table 1, where 10 means that the recommendations proposed by Spanish Society Community Nutrition (SENC) [35] were met.

**Table 2:** Personal, dietetic, and biochemical characteristics according to sex.

	Total (*n* = 1747)	Men (*n* = 771)	Women (*n* = 976)
Age (years; × ± SD)	66.21 ± 10.64	65.49 ± 10.45	66.79 ± 10.76 *
Median (p25–p75)	65 (57–75)	65 (56–74)	66 (57–76)
Educational level (%)			
Very low	1.1	0.4	1.6 *
Low	77	71.1	81.7 *
High	21.9	28.5	16.7 *
Marital status (%)			
Single	5.6	4.5	6.4
Married/in a couple	70.3	85.7	58.2 *
Widow/widower	19.5	7.1	29.3 *
Divorced	4.6	2.6	6.1 *
Smoking habits (%)			
Nonsmokers	54.1	34.8	69.5 *
Ex-smokers	31.5	47.8	18.4 *
Daily smokers	12.7	14.9	11.0 *
Not daily smokers	1.7	2.4	1.0 *
Inflammatory disease (%)	36.5	37.2	36
Arthrosis (%)	28.3	28.4	28.3
Asthma (%)	4.1	4	4.1
COPD (%)	4.6	4.8	4.5
Diabetes (%)	8.5	9.6	7.7
Sleep duration (hours; × ± SD)	8.06 ± 2.73	9.11 ± 3.11	7.22 ± 2.01 ***
7.7GFS (points; × ± SD)	23.42 ± 6.34	20.04 ± 5.30	26.12 ± 5.78 ***
SHEI Score (points; × ± SD)	76.52 ± 8.38	75.08 ± 8.54	77.67 ± 8.08 ***
Need changes/inadequate diet quality ^1^ (%)	57.8	66.2	51.2 *
Neutrophils (1000/mm^3^; × ± SD)	3.86 ± 1.30	4.00 ± 1.33	3.75 ± 1.27 ***
Lymphocytes (1000/mm^3^; × ± SD)	2.06 ± 0.67	2.03 ± 0.70	2.08 ± 0.64 *
NLR (× ± SD)	2.03 ± 0.89	2.15 ± 0.96	1.93 ± 0.82 ***
Inflammation status ^2^ (%)	19.8	17.38	18.1

Abbreviations: COPD, chronic obstructive pulmonary disease; GFS, Global Food Score; SHEI, Spanish Healthy Index; NLR, neutrophil-to-lymphocyte ratio. ^1^ SHEI < 80 points; ^2^ Inflammation status measured by NRL: total: ≥2.6; women: ≥2.4; men: ≥2.8. Significant differences regarding sex * *p* < 0.05, *** *p* < 0.001.

**Table 3:** Anthropometric characteristics according to sex.

	Total (*n* = 1747)	Men (*n* = 771)	Women (*n* = 976)
BMI (kg/m^2^)	28.36 ± 5.60	28.55 ± 4.07	28.20 ± 4.98 *
<22 kg/m^2^ (%)	6	3.7	7.8 *
22–27 kg/m^2^ (%)	35.3	33	37.2
>27 kg/m^2^ (%)	58.7	63.3	55.0 *
WC (cm) (± SD)	97.41 ± 12.12	100.67 ± 10.42	94.79 ± 12.75 ***
Median (p25–p75)	97.33 (89.33–104.50)	100.00 (94.33–106.67)	94.00 (86.17–103.00)
Women: ≥80 cm; Men: ≥94 cm (%)	83.5	76.3	89.2 *
WHtR (× ± SD)	0.60 ± 0.08	0.60 ± 0.06	0.61 ± 0.09 **
Median (p25–p75)	0.60 (0.55–0.65)	0.59 (0.55–0.63)	0.60 (0.55–0.67)
Women: ≥0.53; Men: ≥0.52 (%)	85.7	89.5	82.6 *
WHR (× ± SD)	0.93 ± 0.08	0.97 ± 0.07	0.91 ± 0.08 ***
Median (p25–p75)	0.94 (0.88–0.99)	0.97 (0.93–1.01)	0.90 (0.85–0.96)
Women: >0.85; Men: >1 (%)	53	28.2	73.9 *
Body fat (%) (× ± SD)	34.12 ± 7.67	30.63 ± 6.16	36.96 ± 7.61 ***
Women: 31–33%; Men: 21–25% (%)	86.2	94.6	79.3 *
Women: >33%; Men: >25% (%)	77.5	83.4	72.6 *

Abbreviations: BMI, body-mass index; WC, waist circumference; WHtR, waist-to-height ratio; WHR, waist-to-hip ratio. Significant differences regarding sex * *p* < 0.05, ** *p* < 0.01, *** *p* < 0.001.

**Table 4:** Personal and dietetic data regarding neutrophil-to-lymphocyte ratio (NLR).

	Total (*n* = 1747)		Men (*n* = 771)		Women (*n* = 976)	
	<p80 *n* = 1153	≥p80 *n* = 284	<p80 *n* = 519	≥p80 *n* = 119	<p80 *n* = 629	≥p80 *n* = 170
Age (years; × ± SD)	64.60 ± 10.16	68.70 ± 10.79 ***	63.65 ± 9.89	69.04 ± 10.53 ***	65.28 ± 10.27	68.71 ± 11.03 ***
Median (p25–p75)	64 (56–72)	69 (60–77)	63 (55–71)	70 (61–77)	64 (56–72)	68 (60–79)
Smoking habits (%)						
Nonsmokers	55.3	50.2	36.7	29.4	69	70.4
Ex-smokers	30.3	34	45.3	54.1	19	16.4
Daily smokers	12.6	15.1	15.5	15.6	10.8	12.5
Not daily smokers	1.8	0.8	2.6	0.9	1.2	0.7
Sleep duration (hours; × ± SD)	8.03 ± 2.75	8.06 ± 2.51	9.05 ±3.20	9.00 ± 2.70	7.27 ± 2.00	7.06 ± 1.98
Inflammatory diseases (%)	36.2	38.4	38.3	32.8	35.3	38.8
Arthrosis (%)	27.6	29.9	28.7	24.4	27.5	30.6
Asthma (%)	4.1	4.6	4.4	2.5	4.3	4.1
COPD (%)	4.4	5.6	4.8	5.9	4.3	4.7
Diabetes (%)	8.7	9.5	10.8	6.7	8.1	7.1
GFS (points; × ± SD)	23.56 ± 6.41	22.65 ± 6.26 *	19.89 ± 5.18	19.92 ± 5.88	26.34 ± 5.90	25.22 ± 5.41 *
SHEI Score (points; × ± SD)	76.85 ± 8.08	75.85 ± 9.41	75.33 ± 8.30	74.95 ± 9.51	78.16 ± 7.67	76.20 ± 9.27 *
Need changes/inadequate diet quality ^1^ (%)	56.2	61.3	65.5	64.8	48.5	59.7 *

Abbreviations: COPD, chronic obstructive pulmonary disease; GFS, Global Food Score; SHEI, Spanish Healthy Index. ^1^ SHEI < 80 points. Significant differences in each group: * *p* < 0.05; *** *p* < 0.001.

**Table 5:** Anthropometric data regarding neutrophil-to-lymphocyte ratio (NLR).

	Total (*n* = 1747)		Men (*n* = 771)		Women (*n* = 976)	
	<p80 *n* = 1153	≥p80 *n* = 284	<p80 *n* = 519	≥p80 *n* = 119	<p80 *n* = 629	≥p80 *n* = 170
BMI (kg/m^2^)	28.16 ± 4.46	28.70 ± 4.90	28.45 ± 3.91	28.92 ± 4.61	28.00 ± 4.84	28.22 ± 5.14
<22 kg/m^2^	6.1	5.9	3.4	5.6	7.7	8.8
22–27 kg/m^2^	36.6	33.3	33.3	30.6	39.4	35.1
>27 kg/m^2^	57.3	60.8	63.3	63.9	53	56.1
WC (cm) (× ± SD)	96.53 ± 11.85	99.52 ± 12.20 ***	100.18 ± 10.22	101.82 ± 10.34 *	94.18 ± 12.32	95.49 ± 13.39
Median (p25–p75)	96.67 (88.67–104)	99.5 (91–105)	100 (94–106)	101.58 (95.33–108)	93.67 (85.67–102)	95.33 (87.83–103.17)
Women: ≥80 cm; Men: ≥94 cm (%)	83.4	82.5	75.5	78.3	89.4	87.2
WHtR (× ± SD),	0.60 ± 0.08	0.62 ± 0.08 ***	0.59 ± 0.06	0.61 ± 0.06 ***	0.60 ± 0.08	0.62 ± 0.09 *
Median (p25–p75)	0.59 (0.55–0.64)	0.62 (0.57–0.66)	0.59 (0.55–0.62)	0.61 (0.57–0.64)	0.60 (0.55–0.66)	0.62 (0.56–0.69)
Women: ≥0.53; Men: ≥0.52 (%)	84.8	88.9	89	93.4	82.5	81.2
WHR (× ± SD)	0.93 ± 0.08	0.95 ± 0.08 ***	0.97 ± 0.07	0.98 ± 0.06 *	0.90 ± 0.08	0.91 ± 0.09 *
Median (p25–p75)	0.94 (0.88–0.98)	0.95 (0.90–1.00)	0.97 (0.93–1.01)	0.98 (0.94–1.01)	0.89 (0.85–0.95)	0.91 (0.86–0.98)
Women: >0.85; Men: >1 (%)	53.1	53.6	72	69.8	73.3	75.2
Body fat (%) (× ± SD)	34.08 ± 7.65	34.21 ± 7.68	30.31 ± 6.16	31.68 ± 5.94 *	37.01 ± 7.38	36.73 ± 8.26
Women: 31–33%; Men: 21–25% (%)	85.7	89.5	93.9	97	80.6	77.9
Women: >33%; Men: >25% (%)	76.5	81.5	81.6	88.1	73.2	72.9

Abbreviations: BMI, body-mass index; WC, waist circumference; WHtR, waist-to-height ratio; WHR, waist-to-hip ratio. Significant differences in each group: * *p* < 0.05; *** *p* < 0.001.
